# Why -aVF can be used in STAN as a proxy for scalp electrode-derived signal; reply to comments by Kjellmer et al.

**DOI:** 10.1371/journal.pone.0221220

**Published:** 2019-08-22

**Authors:** Tammo Delhaas, Peter Andriessen, Judith OEH van Laar, Rik Vullings, Ben JM Hermans, Hendrik J. Niemarkt, Reint K. Jellema, Daan RMG Ophelders, Tim GAM Wolfs, Boris W. Kramer, Alex Zwanenburg

**Affiliations:** 1 Department of Biomedical Engineering, Maastricht University, Maastricht, the Netherlands; 2 CARIM School for Cardiovascular Diseases, Maastricht University, Maastricht, the Netherlands; 3 Department of Pediatrics, Máxima Medical Centre, Veldhoven, the Netherlands; 4 Department of Pediatrics, Maastricht University Medical Centre, Maastricht, the Netherlands; 5 Department of Obstetrics and Gynecology, Máxima Medical Centre, Veldhoven, the Netherlands; 6 Signal Processing Systems Group, Department of Electrical Engineering, Eindhoven University of Technology, Eindhoven, the Netherlands; 7 School for Mental Health and Neuroscience, Maastricht University, Maastricht, the Netherlands; University of Washington, UNITED STATES

## Abstract

The conclusion of our recent paper that performance of the STAN device in clinical practice is potentially limited by high false-negative and high false-positive STAN-event rates and loss of ST waveform assessment capacity during severe hypoxemia, evoked comments by Kjellmer, Lindecrantz and Rosén. These comments can be summarized as follows: 1) STAN analysis is based on a unipolar lead but the authors used a negative aVF lead, and they did not validate this methodology; 2) The fetuses used in the study were too young to display the signals that the authors were trying to detect. In response to these comments we now provide both a theoretical and an experimental underpinning of our approach. In an *in vivo* experiment in human we placed several electrodes over the head (simulating different places of a scalp electrode), simultaneously recorded Einthoven lead I and II, and constructed −aVF from these two frontal leads. Irrespective of scalp electrode placement, the correlation between any of unipolar scalp electrode-derived signals and constructed–aVF was excellent (≥ 0.92). In response to the second comment we refer to a study which demonstrated that umbilical cord occlusion resulted in rapid increase in T/QRS ratio that coincided with initial hypertension and bradycardia at all gestational ages which were tested from 0.6–0.8 gestation. The animals of our study were in this gestational range and, hence, our experimental setup can be used to assess STAN’s quality to detect fetal hypoxia. In conclusion, we have clearly demonstrated the appropriateness of using–aVF as a proxy for a scalp electrode-derived signal in STAN in these preterm lambs. Investigation why STAN could not detect relevant ST-changes and instead produced erroneous alarms in our experimental setup is hampered by the fact that the exact STAN algorithm (signal processing and analysis) is not in the public domain.

## Introduction

In our recent paper "*ST waveform analysis for monitoring hypoxic distress in fetal sheep after prolonged umbilical cord occlusion*" [1], we concluded that the performance of the STAN device in clinical practice is potentially limited by high false-negative and high false-positive STAN-event rates and loss of ST waveform assessment capacity during severe hypoxemia. This conclusion evoked comments by Kjellmer, Lindecrantz and Rosén that can be summarized as follows: 1) STAN analysis is based on a unipolar lead but the authors used a negative aVF lead, and they did not validate this methodology; 2) The fetuses used in the study were too young to display the signals that the authors were trying to detect.

## Reply to first comment

Though we did not show any experimental proof for the appropriateness of our methodology in the paper commented on, from a theoretical point of view our method of reconstructing −aVF as a proxy for a unipolar scalp electrode lead cannot be doubted (as shown below). In this rebuttal we will also provide experimental evidence.

Mathematically, augmented limb leads are scaled true unipolar ECG leads. The unipolar foot electrode (VF) would be calculated by:

$$VF=\phi_{f}-\phi_{WCT}=\phi_{f}-\frac{\phi_{f}+\phi_{r}+\phi_{l}}{3}=\frac{2\phi_{f}-\phi_{r}-\phi_{l}}{3}$$ (1)

with *ϕ_f_,ϕ_r_* and *ϕ_l_* the potential recorded at the foot, right arm and left arm respectively and *ϕ_WCT_* the Wilson central terminal. The augmented limb lead aVF is calculated by:

$$aVF=\phi_{f}-\frac{\phi_{r}+\phi_{l}}{2}=\frac{2\phi_{f}-\phi_{r}-\phi_{l}}{2}$$ (2)

So, VF can be calculated from aVF by scaling aVF with 2/3.

$$\frac{2}{3}∙\frac{\left(2\phi_{f}-\phi_{r}-\phi_{l}\right)}{2}=\frac{2\phi_{f}-\phi_{r}-\phi_{l}}{3}$$ (3)

The only difference between aVF and VF is thus the augmented amplitude of aVF. Because aVF and VF represent the “look down” or towards the feet forces, negative aVF and negative VF represents towards the head electrical forces.

Contrary as suggested by Kjellmer et al., we never stated that we used precordial fetal ECG leads. The reference paper by Jellema et al. [2] states ‘Three custom-made electrocardiogram (ECG) shielded electrodes (Cooner Wire Co., Chatsworth, CA, USA) with silver plates (5 mm) were sewn on the chest for fetal heart rate recordings’. The paper by Zwanenburg et al. [3] states ‘Three custom-made silver electrodes with fixed leads (Cooner, Chatsworth, CA) were inserted in the subdermis and fixed with sutures. These electrodes were used for electrocardiography (ECG) and formed an Einthoven triangle’.

**Fig 1:**
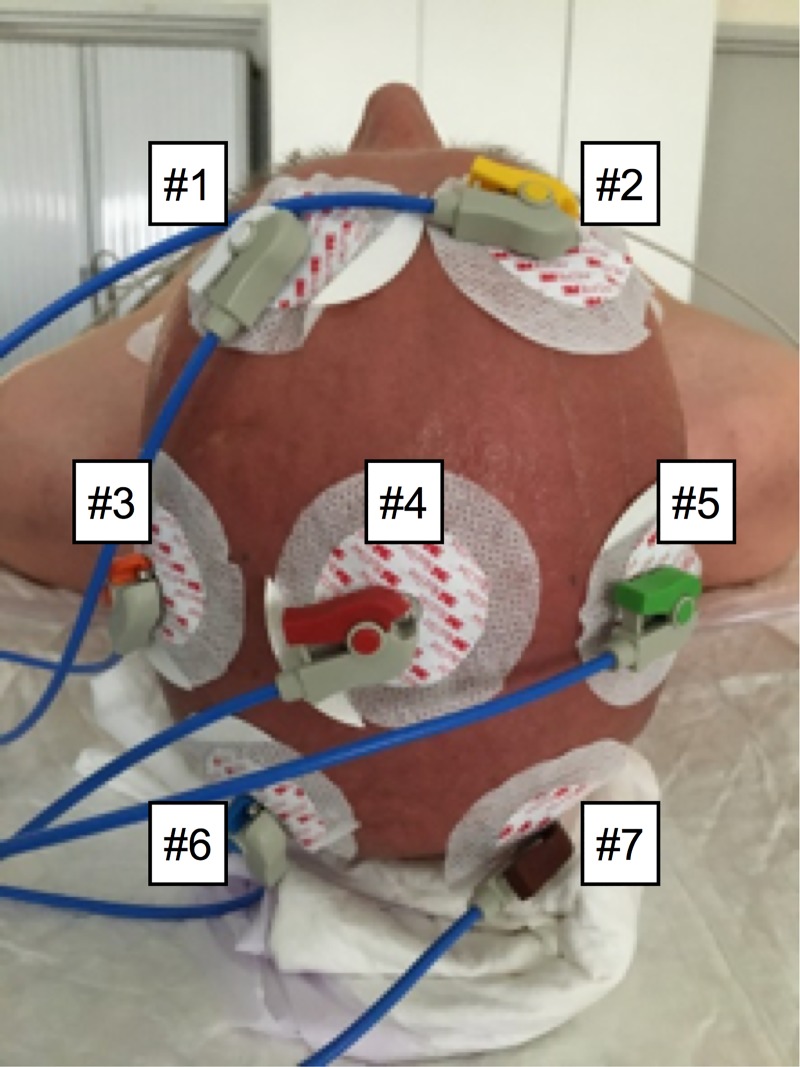
Placement scalp unipolar electrodes while simultaneously recording Einthoven lead I and II.

We demonstrated the validity of our approach to reconstruct −aVF from leads I and II and use it as a proxy for a scalp electrode derived signal in an *in vivo* experiment in which we placed several electrodes over the head (simulating different places of a scalp electrode) and simultaneously recorded Einthoven lead I and II (Fig 1).

**Fig 2:**
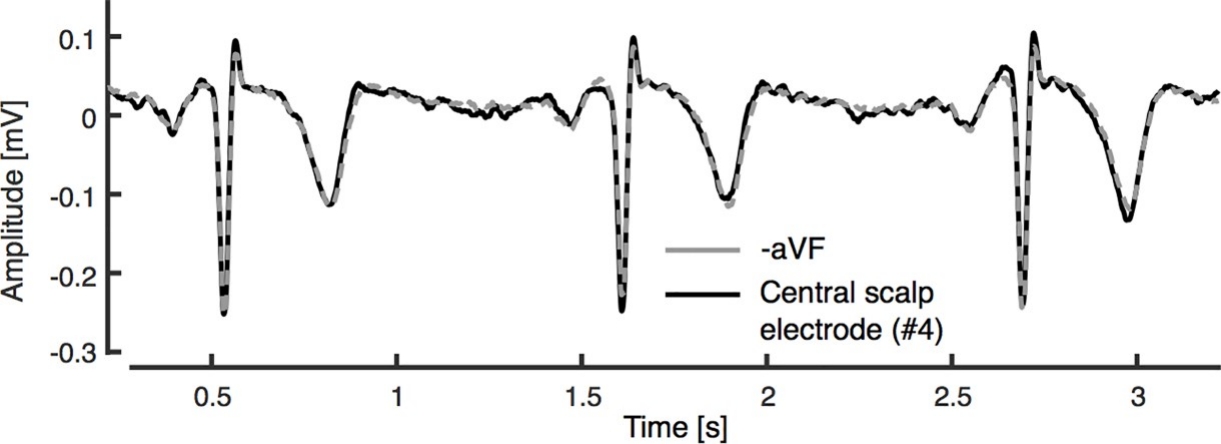
Comparison between constructed −aVF and the central unipolar scalp electrode-derived signal.

Using lead I and II, we constructed −aVF and compared this signal with the unipolar scalp electrode-derived signals (Fig 2). Irrespective of the placement of the scalp electrode there was an excellent correlation (≥ 0.92) between any of unipolar scalp electrode-derived signals and constructed–aVF (Table 1).

**Table 1:** Correlation between −aVF and different electrode positions on the scalp.

Lead	−aVF	Scalp #1	Scalp #2	Scalp #3	Scalp #4	Scalp #5	Scalp #6	Scalp #7
−aVF	1	0.929	0.917	0.976	0.980	0.957	0.983	0.982
Scalp #1	0.929	1	0.994	0.961	0.968	0.944	0.957	0.956
Scalp #2	0.917	0.994	1	0.949	0.961	0.942	0.948	0.949
Scalp #3	0.976	0.961	0.949	1	0.993	0.968	0.996	0.995
Scalp #4	0.980	0.968	0.961	0.993	1	0.979	0.996	0.995
Scalp #5	0.957	0.944	0.942	0.968	0.979	1	0.972	0.971
Scalp #6	0.983	0.957	0.948	0.996	0.996	0.972	1	0.999
Scalp #7	0.982	0.956	0.949	0.995	0.995	0.971	0.999	1

Given the fact that STAN correctly detected heart rate, STAN could properly deal with our–aVF-signal. Conceivably, there must be some other reason why the STAN device produced high false positive event rates during baseline and did not detect T/QRS changes adequately after prolonged fetal hypoxemia.

## Reply to second comment

Both preterm and term ovine fetuses show hypoxia-induced ST waveform changes after umbilical cord occlusion. We refer to the study by Wassink *et al*. [4] which demonstrated that umbilical cord occlusion resulted in a rapid increase in T/QRS ratio that coincided with initial hypertension and bradycardia at all gestational ages which were tested from 0.6–0.8 gestation. The animals of our study were in this gestational range and, hence, our experimental setup can be used to assess STAN’s quality to detect fetal hypoxia.

In response to the remark that one of the authors (R. Vullings) stated in his PhD-thesis that the STAN bipolar lead configuration “was not far from optimal” we dare to say that this statement by Vullings should be read in its context. In his PhD-thesis [5], Vullings considered maximum T-wave amplitude as a measure of signal quality of the T/QRS ratio that is used by STAN. Though he found that the STAN bipolar lead configuration showed the maximum T-wave amplitude, this should not be read as the STAN bipolar lead configuration being optimal for monitoring fetal compromise. Moreover, in his PhD-thesis Vullings stated that results were obtained on two patients only and there was no statistical significance of the findings.

Finally, we want to make a general comment regarding the validity of the methodology used by STAN. The arguments by Kjellmer et al. that they have patented digital processing features and that the STAN FECG analysis features were a key component in the US Food and Drug Administration (FDA) PMA approval process, do in our opinion not rule out the possibility of inadequate ST signal processing by STAN. We would love to investigate and discuss in more detail why STAN was unable to detect relevant ST-changes and instead produced erroneous alarms, but are hampered by the fact that the exact STAN algorithm (signal processing and analysis) is not in the public domain. Because only the definitions used by STAN are in the public domain, the STAN device is in fact a black box.

In our opinion, we have clearly demonstrated the appropriateness of using–aVF as a proxy for a scalp electrode-derived signal in STAN. We also showed that the performance of the STAN device is potentially limited by high false STAN-event rates and loss of ST waveform assessment capacity during severe hypoxemia.
